# Ubrogepant: First Approval

**DOI:** 10.1007/s40265-020-01264-5

**Published:** 2020-02-05

**Authors:** Lesley J. Scott

**Affiliations:** Springer Nature, Private Bag 65901, Mairangi Bay, Auckland, 0754 New Zealand

## Abstract

Ubrogepant (Ubrelvy™) is an orally administered, small molecule, highly-selective, calcitonin gene-related peptide (CGRP) antagonist that was developed by Allergan under license to Merck & Co. as an acute treatment for migraine. In December 2019, ubrogepant received its first global approval in the USA for the acute treatment of migraine (± aura) in adults. This article summarizes the milestones in the development of ubrogepant leading to its first global approval for the acute treatment of migraine (± aura) in adults.

## Ubrogepant (Ubrelvy™): Key points

**Table:** 

A calcitonin gene-related peptide receptor antagonist was being developed by Allergan under license to Merck & Co. for the acute treatment of migraine
Received its first approval on 23 December 2019 in the USA
Approved for use in the acute treatment of migraine (± aura) in adults

## Introduction

Calcitonin gene-related peptide (CGRP), a vasodilatory neuropeptide involved in nociceptive transmission and modulation, and its receptors are widely expressed in central and peripheral regions of the nervous system [1–3]. Extensive evidence supports the important role that CGRP plays in migraine pathophysiology, making CGRP and its receptors a novel therapeutic target for the treatment of migraine. Indeed, the recent development of agents that target CGRP and its receptors represent an important advance in the management paradigm for migraine [1–3].

Ubrogepant (Ubrelvy™), a highly potent, orally administered small molecule, is a CGRP receptor antagonist being developed by Allergan under license from Merck & Co., for the acute treatment of migraine [4]. On the 23 December 2019, the US FDA approved ubrogepant for the acute treatment of migraines (± aura) in adults [5]. It is the first drug in the class of oral CRGP antagonists approved for the acute treatment of migraine [5]. The recommended dosage of oral ubrogepant is 50 mg or 100 mg taken as needed without regard to food [6]. A second dose may be administered at least 2 h after the initial dose if needed, with a maximum dose in a 24-h period of 200 mg. In patients with severe hepatic or renal impairment, the recommended dose is 50 mg; a second dose may be administered at least 2 h after the initial dose if needed [6].

### Company Agreements

In July 2015, Merck & Co. entered into a licensing agreement with Allergan to divest the worldwide rights of small molecule CGRP receptor antagonists, atogepant and ubrogepant. According to the terms of the signed agreement, Allergan will disburse Merck & Co. an upfront payment of $US250 million. Merck & Co. is entitled to receive development and commercial milestone payments, as well as tiered double-digit royalties based on commercialization of the programs. Allergan is completely responsible for development, manufacturing and commercialization upon approval and launch of the products [4].

**Figure:**
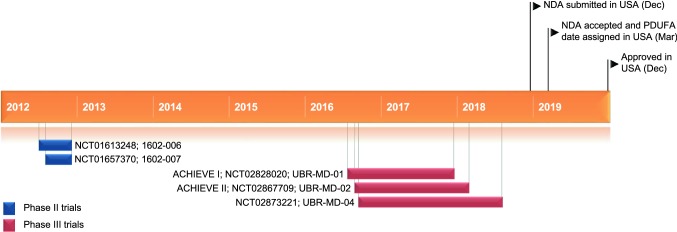
Key milestones in the development of ubrogepant for the acute treatment of migraine. *NDA* New Drug Application, *PDUFA* Prescription Drug User Fee Act

## Scientific Summary

### Pharmacodynamics

Ubrogepant is a potent, highly-selective, competitive CGRP receptor antagonist. In functional assays, ubrogepant exhibited similar high-affinity binding for native CGRP receptors [inhibitory constant (K_i_) 0.067 nmol/L] and for cloned human and rhesus monkey CGRP receptors (K_i_ 0.070 and 0.079 nmol/L at respective cloned receptors). Ubrogepant demonstrated potent inhibition of the human α-CGRP-stimulated cyclic AMP response in human CGRP receptor-expressing HEK293 cells (50% inhibitory concentration of 0.08 nmol/L). Relative to other receptors in the calcitonin receptor family, ubrogepant exhibited highly selective antagonist activity against CGRP receptors [7].

Therapeutic concentrations of ubrogepant did not induce major vasoconstrictor effects in cultured human coronary, cerebral and middle meningeal artery in vitro. Ubrogepant exhibited competitive inhibition of α-CGRP-induced relaxations, with antagonism of CGRP-induced relaxation more potent for cranial (middle meningeal and cerebral) than coronary arteries [8].

At doses twice the maximum recommended daily dose, ubrogepant does not prolong the QT interval to any clinically relevant extent [6], based on results of thorough QT study in healthy adults [9].

### Pharmacokinetics

Ubrogepant exhibits dose-proportional pharmacokinetics and is rapidly absorbed after oral administration, with peak plasma concentrations attained at ≈ 1.5 h. There are no clinically relevant effects of food on the pharmacokinetics of ubrogepant. The drug is 87% bound to plasma protein in vitro. After a single oral dose, the mean apparent central volume of distribution of ubrogepant is ≈ 350 L [6].

The primary route of metabolism is via CYP3A4, with the parent compound and two glucuronide conjugate metabolites the most prevalent circulating components. The glucuronide metabolites were ≈ 6000-fold less potent in the CGRP receptor binding assay and thus, are not expected to contribute to the pharmacological activity of ubrogepant. The elimination half-life of ubrogepant is ≈ 5 to 7 h. Ubrogepant is primarily eliminated via the biliary/faecal route, with renal elimination a minor route (42% and 6% of a radiolabeled dose recovered as parent compound in the faeces and urine, respectively) [6].

**Figure:**
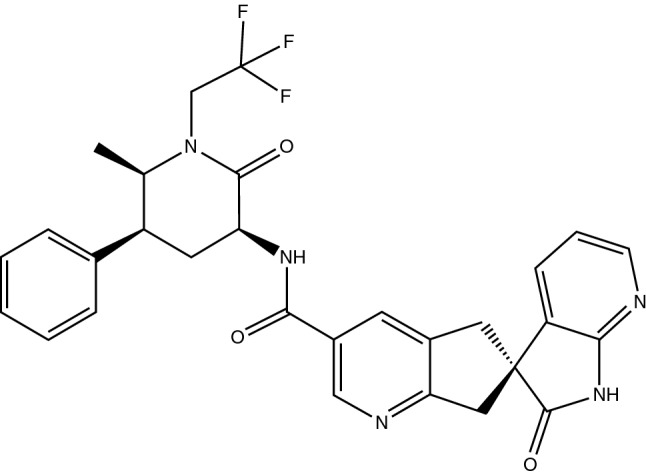
Chemical structure of ubrogepant

There were no clinically relevant effects of age, sex, race, bodyweight, mild or moderate renal impairment, and mild or moderate hepatic impairment on the pharmacokinetics of ubrogepant, based on a population pharmacokinetic (PPK) analyses. Patients with severe renal impairment (estimated glomerular filtration rate < 30 mL/min) have not been studied; dose adjustment is recommended based on absorption, distribution, metabolism and elimination (ADME) information and a conservative assumption that severe renal impairment is unlikely to cause a more than twofold increase in exposure to ubrogepant. No dosing recommendations can be made for patients with end-stage renal disease (creatinine clearance < 15 mL/min). In patients with severe hepatic impairment (Child–Pugh Class C), exposure to ubrogepant increased by 115%; dosage adjustments are required in this population [6].

In vitro, ubrogepant does not inhibit CYP1A2, CYP2B6 or CYP3A4 and is a weak inhibitor of CYP2C8, CYP2C9, CYP2D6, CYP2C19, MAO-A and UGT1A1; this inhibition is not expected to be clinically relevant. At clinically relevant concentrations, ubrogepant is not an inducer of CYP1A2, CYP2B6 or CYP3A4. The drug is a substrate of BCRP and P-glycoprotein (P-gp) transporters in vitro; thus, use of inhibitors of BCRP and/or P-gp may increase exposure to ubrogepant, with dose adjustments of ubrogepant recommended when coadministered with BCRP and/or P-gp only inhibitors (e.g. quinidine, carvedilol, eltrombopag, curcumin). Ubrogepant is a weak substrate of OATP1B1, OATP1B3 and OAT1 transporters, but is not a substrate for OAT3 in vitro. It does not inhibit P-gp, BCRP, BSEP, MRP3, MRP4, OAT1, OAT3 or NTCP transporters, but is a weak inhibitor of OATP1B1, OATP1B3 and OCT2 transporters. No clinically relevant drug interactions are expected with these transporters, except for BCRP and P-gp inhibitors [6].

In healthy volunteers, no clinically relevant pharmacokinetic interactions were observed when ubrogepant was co-administered with oral contraceptives (containing norgestimate and ethinyl estradiol) [10], acetaminophen [11], naproxen [11], sumatriptan [12] or esomeprazole [6]. Coadministration of ubrogepant with ketoconazole (strong CYP3A4 inhibitor) resulted in a significant increase in exposure to ubrogepant; hence, concomitant use of ubrogepant with strong CYP3A4 inhibitors (e.g. ketoconazole, itraconazole, clarithromycin) is contraindicated [6]. Concomitant use of ubrogepant with verapamil (a moderate CYP3A4 inhibitor) increased exposure to ubrogepant; adjustment of the ubrogepant dose is recommended with concomitant use of ubrogepant and moderate CYP3A4 inhibitors (e.g. cyclosporine, ciprofloxacin, fluconazole, fluvoxamine, grapefruit juice). Exposure to ubrogepant was significantly reduced when the drug was coadministered with rifampin (a strong CYP3A4 inducer); thus, concomitant use of ubrogepant with strong CYP3A4 inducers (e.g. rifampin, phenytoin, barbiturates, St. John’s wort) should be avoided. Dose adjustment of ubrogepant is recommended when the drug is coadministered with moderate or weak CYP3A4 inducers [6].

**Table:** **Features and properties of ubrogepant**

Alternative names	Ubrogepant; Ubrelvy™; MK-1602
Class	Amides, antimigraines, fluorine compounds, small molecules, spiro compounds
Mechanism of Action	Calcitonin gene-related peptide receptor antagonists
Route of Administration	Oral
Pharmacodynamics	Potent, highly-selective, competitive calcitonin gene-related peptide receptor antagonist
Pharmacokinetics	Rapidly absorbed; primarily metabolized by CYP3A4; elimination half-life ≈ 5 to 7 h; mainly eliminated via the biliary faecal route, with minor elimination via the renal route
Adverse events	
Most frequent (≥ 2% and > placebo)	Nausea and somnolence; generally well tolerated
ATC codes	
WHO ATC code	N02C (antimigraine preparations)
EphMRA ATC code	N2C (anti-migraine preparations)
Chemical Name	(3S)-N-[(3S,5S,6R)-6-methyl-2-oxo-5-phenyl-1-(2,2,2-trifluoroethyl)piperidin-3-yl]-2-oxospiro[1H-pyrrolo[2,3-b]pyridine-3,6′-5,7-dihydrocyclopenta[b]pyridine]-3′-carboxamide

### Therapeutic Trials

In pivotal randomized, double-blind, multicentre, phase III trials (ACHIEVE I [13] and II [14]) in adults with moderate to severe migraine, recommended doses of ubrogepant (50 or 100 mg) were significantly more effective than placebo for the proportion of patients achieving the co-primary endpoints of freedom from pain 2 h after the initial dose and the absence of the most bothersome symptom (MBS; patient-specified MBS out of photophobia, phonophobia or nausea) associated with migraine at 2 h postdose. In ACHIEVE I, significantly (all *p* ≤ 0.002) more patients in the ubrogepant 50 mg and 100 mg groups experienced freedom from migraine pain at 2 h postdose than placebo recipients (19.2% and 21.2% vs 11.8%, respectively; *n* = 422, 448 and 456), with absence of the most bothersome migraine-associated symptom at 2 h postdose achieved by 38.6%, 37.7% and 27.8% of patients (both *p* = 0.002 vs placebo; *n* = 420, 448 and 454) [13]. In ACHIEVE II, significantly more patients in the ubrogepant 50 mg than placebo group experienced freedom from pain at 2 h postdose (21.8 vs 14.3%; *p* = 0.01; *n* = 464 and 456) and absence of the MBS associated with migraine at 2 h postdose (38.9 vs 27.4%; *p* = 0.01; *n* = 463 and 456) [14].

Secondary endpoints for pain relief also generally favoured ubrogepant 50 or 100 mg over placebo treatment, including the percentage of patients with pain relief at 2 h (≈ 61.6 vs ≈ 48.7% of patients in individual treatment groups across trials; all *p* ≤ 0.01 vs placebo), sustained pain relief from 2 to 24h (≈ 37.0 vs ≈ 20.9%; all *p* ≤ 0.01 vs placebo) and sustained pain freedom from 2 to 24 h (≈ 14.2 vs ≈ 8.4%; significance not evaluated for ubrogepant 50 mg vs placebo in ACHIEVE I in accordance with the hierarchical statistical testing plan; *p* ≤ 0.01 vs placebo for ubrogepant 100 mg in ACHIEVE I and ubrogepant 50 mg in ACHIEVE II) [13, 14]. In both studies, the incidence of photophobia and phonophobia was reduced after treatment with ubrogepant 50 mg or 100 mg compared with placebo [6, 13, 14].

Based on pooled analyses of the 50 mg dose across ACHIEVE I and II, the onset of pain relief was significant at 1 h in ubrogepant versus placebo recipients [odds ratio (OR) 1.30; *p* = 0.01] [15] and ubrogepant was effective in patients for whom triptans were ineffective [16] (abstracts). In patients who took an optional per-protocol specified second dose of ubrogepant 50 mg or placebo (re-randomization of ubrogepant 50 mg recipients) after an inadequate response to the initial dose of ubrogepant 50 mg, significantly more ubrogepant than placebo recipients achieved freedom from pain 2 h after the second dose (34 vs 19%; OR 2.85; *p* < 0.0001; *n* = 156 and 131), based on pooled data (abstract) [17]. In patients achieving freedom from pain 2 h after the initial dose and who took an optional second dose, this response rate was also significantly better in the ubrogepant than placebo group (55 vs 33%; OR 2.85; *p* < 0.0001; *n* = 75 and 57) [17].

In ACHIEVE I and II, participants were aged 18–75 years, had at least a 1-year history of migraine (± aura) that met specified International Classification of Headache Criteria, had migraine onset before 50 years of age and a history of 2–8 migraines with moderate to severe headache pain in each of the 3 months prior to screening. Patients also had to have a history of migraine typically lasting 48–72 h if treated successfully or unsuccessfully and migraine episodes separated by ≥ 48 h of headache pain freedom [13, 14]. Patients were randomized to ubrogepant 50 mg or 100 mg or placebo in ACHIEVE I [13] and to ubrogepant 50 mg or placebo in ACHIEVE II (also had an ubrogepant 25 mg arm which is not discussed) [14]. In both trials, participants were instructed to treat a migraine with moderate to severe headache pain intensity. A second dose of study medication (ubrogepant or placebo) or the patient’s usual acute treatment for migraine (included acetaminophen, NSAIDs, opioids, anti-emetics or triptans [14]) was allowed between 2 to 48 h after the initial treatment for non-responding or recurrent migraine headache [13, 14]. Among patients who selected a MBS, photophobia was selected as the MBS by 56% of patients, phonophobia by 24% and nausea by 19% [6].

Evidence from ACHIEVE I and II are supported by data from a randomized, dose-finding, double-blind, multicentre phase IIb trial (NCT01613248) in adults with migraine [18]. An integrated exposure–response modeling analysis of this phase IIb study and another phase IIb study (NCT01657370) predicted that a dose of ubrogepant 25 mg or higher was likely to achieve significantly better efficacy than placebo with desirable efficacy levels. These data supported the ubrogepant dose selection utilized in the pivotal phase III trials [19].

The beneficial effects of ubrogepant (50 or 100 mg doses) observed in the short-term ACHIEVE trials were maintained during the 1-year open-label extension study (NCT02873221; *n* = 808 modified intent to treat participants) [abstract] [20]. Over the 1-year period, pain freedom at 2 h after the initial dose occurred in ≈ 24% of ubrogepant-treated (50 or 100 mg) migraine attacks and pain relief at 2 h postdose occurred in ≈ 67% of ubrogepant-treated attacks. During this 1-year period, 21,454 migraine attacks were treated with 31,968 doses of ubrogepant and an optional second dose of ubrogepant was taken for ≈ 35% of attacks. Participants were randomized equally to open-label usual care or ubrogepant 50 mg or 100 mg, as required for migraine attacks [20].

**Table:** **Key clinical trials of ubrogepant for the acute treatment of migraine in adults (Allergan)**

Drug(s)	Phase	Status	Location(s)	Identifier
Ubrogepant vs placebo	II	Completed	USA	NCT01657370; 1602-007
Ubrogepant vs placebo	II	Completed	USA	NCT01613248; 1602-006
Ubrogepant vs placebo	III	Completed	USA	NCT02828020; ACHIEVE I; UBR-MD-01
Ubrogepant vs placebo	III	Completed	USA	NCT02867709; ACHIEVE II; UBR-MD-02
Ubrogepant vs placebo	III	Completed	USA	NCT02873221; UBR-MD-04

### Adverse Events

Ubrogepant was generally well tolerated in adults with migraine participating in phase I [21, 22], II [18] and III [13, 14] trials. Based on pooled data from ACHIEVE I and II, the most commonly (frequency ≥ 2% and at a frequency greater than placebo) reported adverse reactions occurring in the ubrogepant 50 mg (*n* = 954), ubrogepant 100 mg (*n* = 485) and placebo (*n* = 984) groups were nausea (2, 4 and 2% of patients, respectively), somnolence (2, 3 an 1%; includes sedation and fatigue) and dry mouth (< 1, 2 and 1%) [6]. Within the 48-h period postdose, 107 of 954 (11.5%) ubrogepant-treated (50 mg dose) patients and 113 of 984 (11.2%) placebo recipients experienced ≥ 1 treatment-emergent adverse event (TEAE), with no serious TEAEs reported in either group during this 48-h period (pooled ACHIEVE data; abstract) [23]. Within 30 days of any dose, ≥ 1 TEAE was reported by 27.1% and 22.9% of ubrogepant and placebo recipients, with 9.4% and 8.9% of these TEAEs considered by the investigator to be treatment-related. Of the three serious TEAEs reported, none were considered treatment-related. Of the five cases of aspartate transaminase (AST)/alanine transaminase (ALT) levels ≥ 3 × the upper limit of normal (ULN) reported in the ubrogepant 50 mg group, all were judged by an independent panel of liver experts blinded to treatment to be unlikely to be related to study treatment [23].

During the 48-h and 30-day postdose periods, there was no evidence of an increased risk of adverse events in ubrogepant recipients based on the presence or absence of cardiovascular (CV) risk factors at baseline in ACHIEVE I and II (pooled data), regardless of whether patients were categorized as having moderate to high, low or no CV risk (abstract) [24].

No safety concerns were identified during longer-term acute treatment of migraine attacks with ubrogepant 50 mg or 100 mg, as required, in the 1-year extension study [25]. The safety population comprised 404, 409 and 417 patients in the ubrogepant 50 mg, ubrogepant 100 mg and usual care groups, respectively; over the 1-year study period, 21,454 migraine attacks were treated with 31,968 doses of ubrogepant. TEAEs were reported by 66.3% and 72.6% of patients in the ubrogepant 50 mg and 100 mg groups during this period, with treatment-related adverse events reported by 10.4% and 10.5% of patients. One patient in the ubrogepant 50 mg group experienced a serious adverse event (sinus tachycardia) that was considered to be treatment-related by the investigator; this patient had a history of supraventricular tachycardia with ablation. Of the 20 cases of AST/ALT levels ≥ 3 × ULN reported across treatment groups, two cases in the ubrogepant 50 mg group were adjudicated by an independent panel of liver experts blinded to treatment to be possibly treatment-related and one case in the ubrogepant 100 mg group was judged to be probably related (confounding factors were noted in this case). There were no cases of Hy’s Law [25].

### Ongoing Clinical Trials

There are currently no ongoing studies evaluating the efficacy of ubrogepant in the acute treatment of migraine. In September 2019, a randomized, open-label, phase I trial (NCT04179474) was initiated to evaluate the potential for a pharmacokinetic interaction and the safety and tolerability of ubrogepant when ubrogepant and erenumab or galcanezumab are coadministered (estimated completion date December 2019).

## Current Status

On the 23 December 2019 [5], ubrogepant received its first global approval in the USA for the acute treatment of migraine (± aura) in adults [6].
