# Correction to: Ubrogepant: First Approval

**DOI:** 10.1007/s40265-020-01292-1

**Published:** 2020-03-12

**Authors:** Lesley J. Scott

**Affiliations:** Springer Nature, Private Bag 65901, Mairangi Bay, Auckland, 0754 New Zealand

## Correction to: Drugs (2020) 80:323–328 10.1007/s40265-020-01264-5

The article Ubrogepant: First Approval, written by Lesley J. Scott, was originally published published Online First without Open Access. After publication online Allergan Sales, LLC requested that the article be Open Choice to make the article an open access publication. Post-publication open access was funded by Allergan Sales, LLC. The article is forthwith distributed under the terms of the Creative Commons Attribution 4.0 International License (http://creativecommons.org/licenses/by/4.0/), which permits use, duplication, adaptation, distribution and reproduction in any medium or format, as long as you give appropriate credit original author(s) and the source, provide a link to the Creative Commons licence and indicate if changes were made.

The original article has been corrected.

